# SGLT2 Inhibitors Are Associated With Suppression of the Sema3A/NRP1/Plexin‐A1 Signaling Axis in Postmenopausal Women With Type 2 Diabetes: Evidence From Propensity Score‐Matched Analysis

**DOI:** 10.1111/1753-0407.70262

**Published:** 2026-08-26

**Authors:** Sertas Erarslan, Burcu Cosgun Bora

**Affiliations:** ^1^ Department of Internal Medicine, Faculty of Medicine Kütahya Health Sciences University Kutahya Turkey

## Abstract

Propensity score‐matched comparison of 23 SGLT2 inhibitor users and 23 non‐users revealed that SGLT2 inhibitor use was associated with significantly lower Neuropilin‐1 (NRP1) levels (74.25 vs. 87.61 ng/mL; *p* = 0.015) and a higher Sema3A/NRP1 ratio (0.14 vs. 0.12; *p* = 0.006), indicating functional impairment of the Sema3A/NRP1/Plexin‐A1 bone signaling axis with compensatory Sema3A upregulation. Classical bone turnover markers (P1NP, CTX, coupling index) and DXA‐derived BMD did not significantly differ between groups. These findings suggest that SGLT2 inhibitors may perturb the Sema3A/NRP1/Plexin‐A1 axis before detectable changes in conventional markers, offering a novel mechanistic perspective to explain the inconsistency between existing meta‐analyses on SGLT2 inhibitor bone safety.

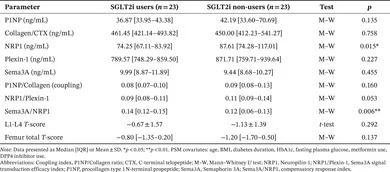

To the Editor,

Sodium‐glucose cotransporter‐2 (SGLT2) inhibitors are widely used in postmenopausal women with type 2 diabetes mellitus (T2DM), yet their effects on bone metabolism remain controversial. Recent meta‐analyses report conflicting results: one (25 RCTs, *n* = 22 828) found significantly increased parathyroid hormone (PTH) and bone resorption marker CTX [1], while another (20 RCTs, *n* = 12 764) found no significant impact on bone turnover markers or BMD [2]. This inconsistency underscores the need for mechanistic studies exploring pathways beyond classical markers.

We recently conducted a cross‐sectional study examining the effect of glycemic variability on the Semaphorin 3A (Sema3A)/Neuropilin‐1 (NRP1)/Plexin‐A1 signaling axis and bone turnover in 126 postmenopausal women with T2DM. As part of this study, we used propensity score matching (PSM) to evaluate the independent effect of SGLT2 inhibitor use on this novel bone signaling axis—to our knowledge, the first such investigation in postmenopausal diabetic women.

The Sema3A/NRP1/Plexin‐A1 axis is critical for bone homeostasis. Sema3A binds NRP1 to suppress osteoclastogenesis via Plexin‐A1/TREM2/DAP12 inhibition [3, 4]. NRP1 suppression disrupts this signaling, activating osteoclasts. The NRP1/Plexin‐1 ratio reflects Sema3A signal transduction efficacy, while Sema3A/NRP1 reflects compensatory Sema3A upregulation.

## 1 Methods

Among 126 participants, 100 (79.4%) used SGLT2 inhibitors. We performed 1:1 PSM controlling for age, BMI, diabetes duration, HbA1c, fasting plasma glucose, metformin, and DPP4 inhibitor use (23 matched pairs; caliper 0.20 × SD logit‐PS). Post‐matching covariate balance was confirmed.

## 2 Results

After PSM, the two groups were well balanced for all covariates (all *p* > 0.05). The results of bone turnover marker comparisons are presented in Table 1.

**TABLE 1:** Bone turnover markers in SGLT2 inhibitor users versus non‐users after propensity score matching (*n* = 23 pairs).

Parameter	SGLT2i users (*n* = 23)	SGLT2i non‐users (*n* = 23)	Test	*p*
P1NP (ng/mL)	36.87 [33.95–43.38]	42.19 [33.60–70.69]	M–W	0.135
Collagen/CTX (ng/mL)	461.45 [421.14–493.82]	450.00 [412.23–541.27]	M–W	0.758
NRP1 (ng/mL)	74.25 [67.11–83.92]	87.61 [74.28–117.01]	M–W	0.015*
Plexin‐1 (ng/mL)	789.57 [748.29–859.50]	871.71 [759.71–939.64]	M–W	0.227
Sema3A (ng/mL)	9.99 [8.87–11.89]	9.44 [8.68–10.27]	M–W	0.455
P1NP/Collagen (coupling)	0.08 [0.07–0.10]	0.09 [0.08–0.13]	M–W	0.160
NRP1/Plexin‐1	0.09 [0.08–0.11]	0.11 [0.09–0.14]	M–W	0.053
Sema3A/NRP1	0.14 [0.12–0.15]	0.12 [0.06–0.13]	M–W	0.006**
L1‐L4 *T*‐score	−0.67 ± 1.57	−1.13 ± 1.39	*t*‐test	0.292
Femur total *T*‐score	−0.80 [−1.35–0.20]	−1.20 [−1.70–0.50]	M–W	0.137

*Note:* Data presented as Median [IQR] or Mean ± SD. *p < 0.05; **p < 0.01. PSM covariates: age, BMI, diabetes duration, HbA1c, fasting plasma glucose, metformin use, DPP4 inhibitor use.

Abbreviations: Coupling index, P1NP/Collagen ratio; CTX, C‐terminal telopeptide; M–W, Mann–Whitney *U* test; NRP1, Neuropilin‐1; NRP1/Plexin‐1, Sema3A signal transduction efficacy index; P1NP, procollagen type 1 N‐terminal propeptide; Sema3A, Semaphorin 3A; Sema3A/NRP1, compensatory response index.

SGLT2 inhibitor users had significantly lower NRP1 levels (74.25 [67.11–83.92] vs. 87.61 [74.28–117.01] ng/mL; *p* = 0.015) and higher Sema3A/NRP1 ratios (0.14 [0.12–0.15] vs. 0.12 [0.06–0.13]; *p* = 0.006), indicating compensatory upregulation of Sema3A. The NRP1/Plexin‐1 ratio trended lower in SGLT2 inhibitor users (0.09 vs. 0.11; *p* = 0.053). Classical markers (P1NP, CTX, coupling index) and DXA‐derived BMD did not significantly differ.

## 3 Discussion

These findings suggest that SGLT2 inhibitor use functionally impairs the Sema3A/NRP1/Plexin‐A1 axis before detectable changes in classical bone markers or BMD—potentially explaining inconsistencies between meta‐analyses.

The mechanism by which SGLT2 inhibitors may suppress NRP1 is not fully established. Proposed pathways include glucosuria‐induced calciuria, leading to secondary hyperparathyroidism; alterations in fibroblast growth factor‐23 (FGF‐23) signaling; and modulation of the renin‐angiotensin‐aldosterone system, all of which may indirectly influence osteoblast‐derived Sema3A signaling [1, 5]. Furthermore, SGLT2 inhibitors have been shown to activate AMP‐activated protein kinase (AMPK) and reduce oxidative stress, effects that may independently modulate axon guidance molecule expression, including semaphorins [6].

The compensatory elevation of Sema3A/NRP1 may represent an adaptive response; however, progressive NRP1 suppression with prolonged exposure could eventually overwhelm this compensation, consistent with the elevated CTX observed by Wang et al. [1].

Limitations include the cross‐sectional design, modest matched sample (*n* = 23 pairs), and unavailability of data on specific SGLT2 inhibitor molecules and treatment duration.

In conclusion, SGLT2 inhibitor use is associated with NRP1 suppression and functional impairment of the Sema3A/NRP1/Plexin‐A1 bone homeostasis axis in postmenopausal women with T2DM, introducing a novel mechanistic perspective to the ongoing bone safety debate.

## Author Contributions

S.E.: study design, data collection, statistical analysis, manuscript writing and editing. B.C.B.: study design, data collection, manuscript writing. All authors read and approved the final manuscript.

## Funding

The authors have nothing to report.

## Ethics Statement

The study was approved by the Kütahya Health Sciences University Ethics Committee.

## Conflicts of Interest

The authors declare no conflicts of interest.

## Data Availability

The data that support the findings of this study are available on request from the corresponding author. The data are not publicly available due to privacy or ethical restrictions.
